# Effects of Prompting in Reflective Learning Tools: Findings from Experimental Field, Lab, and Online Studies

**DOI:** 10.3389/fpsyg.2016.00820

**Published:** 2016-05-31

**Authors:** Bettina Renner, Michael Prilla, Ulrike Cress, Joachim Kimmerle

**Affiliations:** ^1^Leibniz-Institut fuer Wissensmedien/Knowledge Media Research CenterTuebingen, Germany; ^2^Ruhr University, BochumGermany; ^3^Department of Psychology, University of TuebingenTuebingen, Germany

**Keywords:** reflection, prompting, informal learning, instruction, experiment

## Abstract

Reflective learning is an important type of learning both in formal and informal situations—in school, higher education, at the workplace, and in everyday life. People may benefit from technical support for reflective learning, in particular when supporting each other by reflecting not only upon their own but also upon other people’s problems. We refer to this collective approach where people come together to think about experiences and find solutions to problems as “collaborative reflection.” We present three empirical studies about the effects of prompting in reflective learning tools in such situations where people reflect on others’ issues. In Study 1 we applied a three-stage within-group design in a field experiment, where 39 participants from two organizations received different types of prompts while they used a reflection app. We found that prompts that invited employees to write down possible solutions led to more comprehensive comments on their colleagues’ experiences. In Study 2 we used a three-stage between-group design in a laboratory experiment, where 78 university students were invited to take part in an experiment about the discussion of problems at work or academic studies in online forums. Here we found that short, abstract prompts showed no superiority to a situation without any prompts with respect to quantity or quality of contributions. Finally, Study 3 featured a two-stage between-group design in an online experiment, where 60 participants received either general reflection instructions or detailed instructions about how to reflect on other people’s problems. We could show that detailed reflection instructions supported people in producing more comprehensive comments that included more general advice. The results demonstrate that to increase activity and to improve quality of comments with prompting tools require detailed instructions and specific wording of the prompts.

## Introduction

Many learning processes do not take place in formal educational situations but happen through informal everyday learning. This is especially true at the workplace where training courses are by far not the only opportunity for people to learn and acquire knowledge. A particular opportunity for learning at the workplace is through *reflection* upon past work-related experiences (Daudelin, 1996; Knipfer et al., 2013). Reflection has been criticized for being a fuzzy concept the dimensions of which are not always clear (e.g., Davis, 2006; Procee, 2006; Quinton and Smallbone, 2010; Clarà, 2014). It is beyond the scope of this article to discuss all of the potential underlying epistemological considerations or the multiple philosophical theories behind the concept of reflection (e.g., Wertheimer, 1971; Dewey, 1986; Schön, 1987). Instead, we refer interested readers to pertinent publications, such as the considerations by Procee (2006) or Clarà (2014). To reach a minimal consensus on what reflection is, however, and to provide a working conceptualization for this article, we argue that reflection may be regarded as a process in which people remember an experience they have had, think about it, brood over it, and then re-evaluate the experience, thereby often gaining new perspective and new insights about the experience (Boud et al., 1985; Cardador, 2014). The function of reflection is to bring coherence to a situation that was at first incoherent or confusing (Clarà, 2014; see also Dewey, 1986). Reflection should create opportunities for learning (Kinsella, 2001; Procee, 2006). Learning-related knowledge and skills may be outcomes of reflection, which in turn may inform further learning activities (Ertmer and Newby, 1996). Often, but not always, reflection processes result in conclusions that have direct consequences for subsequent actions (Schön, 1987; Clarà, 2014).

In previous research as well as in theoretical and conceptual considerations reflection has largely been conceptualized as an individual process (e.g., Daudelin, 1996). Nevertheless, reflection can be engaged in not only individually, it may also have a strong social dimension (Procee, 2006). Other people may serve as “catalysts” for reflection (Daudelin, 1996, p. 39), as they may nudge or inspire reflection. We argue, however, that the role of other people in the reflection process can even go beyond that of being catalysts. People may explicitly come together in order to reflect jointly upon their problems. For such cases of explicit teamwork with the purpose of reflective learning we use the term *collaborative reflection* (Prilla and Renner, 2014; Prilla, 2015). In collaborative reflection people may provide feedback to others about their respective experiences and reflections (Wopereis et al., 2010). Accordingly, the advantage of collaborative reflection is that people can benefit from the experiences, challenges, and solutions of others. Together they may come up with approaches and aspects they would not have developed when reflecting all by themselves (Raelin, 2001; Høyrup, 2004). Reflection in team sessions needs time, however, which often is a scarce commodity in people’s daily work routines. So a viable approach to sharing experiences with colleagues is to collect them in a shared database. In this way, anyone who faces a particular challenge or wants to share a work experience with their colleagues can contribute it into the shared database. Others can then comment on the situation, evaluate it, or provide advice whenever they wish. These comments and pieces of advice can be taken up, in turn, by the original contributors, or by third parties, creating a common and concerted reflective processing of the issues at hand.

The process of collaborative reflection, that is, collecting contributions from several people, coordinating and combining them, needs specific support; fertile knowledge exchange does not happen by just providing people with a shared tool (Kimmerle et al., 2011; Kimmerle and Cress, 2013). This applies both to many organizational settings where people may use knowledge management systems for purposes of exchanging knowledge and sharing experiences (Kimmerle and Cress, 2009; Wang and Noe, 2010; Kump et al., 2015) and to several online settings such as Internet forums (Joyce and Kraut, 2006; Oliveira et al., 2011; Kimmerle et al., 2012, 2013). In both cases the provided tools allow one to document one’s own experiences but also to ask for and receive help and advice. Online forums are often also applied by organizations, which then are confronted with the task of motivating contributions (Liang et al., 2012; Phang et al., 2015). Usually, users of such tools are quite aware of both of these goals (sharing experiences and receiving feedback on these experiences including potential solution suggestions). However, they can decide on their own in what way and how strongly they want to contribute. In any case, high overall activity is crucial and needs to be fostered. Additionally, deeper levels of reflection such as the documentation of learning and change tend not to develop automatically (Prilla and Renner, 2014). To date, however, there has not been much research on how to provide computer-support for collaborative reflection (for some exceptions see Lin et al., 1999; Lee, 2005; Scott, 2010; Prilla et al., 2013b). Lin et al. (1999) have suggested that technology could play a very important role in facilitating reflection, both individually and collaboratively. These authors provide a theoretical framework that presents several ways of supporting reflection with the help of technology. One of these approaches is the application of *prompts*.

Prompts are a means of scaffolding (Pea, 2004) that may support reflection by guiding the process with leading questions (Lee, 2005; Van den Boom et al., 2007). Prompting has been shown in previous studies to be a suitable method to stimulate reflection processes (e.g., Sobral, 2000; Van den Boom et al., 2004). Such prompts may consist of several (more or less specific) questions that are presented to users while they deal with a reflection tool. Those questions may have different purposes. One purpose is to encourage people to write down their experiences and help them to structure these contributions. In order to support the collaborative side of reflection, prompts can also be presented at a later point when people read through the contributions of others. In this case, prompts are supposed to foster comments that help the authors of the original notes to ponder on the problems that crop up and to solve them (see also Wopereis et al., 2010).

A particular challenge here is to make people comment on others’ contributions in the first place. So the initial goal of prompting collaborative reflection is to stimulate writing *per se* and request users to *provide comprehensive comments*. In addition to preferably elaborate contributions, people should also be as responsive as possible to the particular problem (Nelson, 1999): they should *give advice* to those seeking help, which is particularly relevant in collaborative reflection (Goldsmith and Fitch, 1997; Morrow, 2006). They should give advice both in terms of *concrete solution suggestions* that are targeted directly to the described problem and in terms of *general solution suggestions* that are also applicable in similar situations and therefore would be helpful for a wider range of problems (e.g., Bokhorst et al., 2014; Moskaliuk et al., 2016). So in the research presented here we focused on prompts that would stimulate participants to reflect upon the contributions of others and to draft comprehensive and appropriate comments. In three experiments, which we conducted in diverse settings and with diverse samples of participants, we examined the effects of different types of prompts on users’ contributions. In line with our reasoning above, in all of the studies we captured (1) how comprehensively people commented on others’ contributions, (2) how often they gave concrete advice, and (3) how often they gave general advice.

In Study 1 we used a reflection tool that had already been applied in practice. For this field study with employees we supplemented this tool with two types of prompts (problem prompts and solution prompts). In Study 2, we provided participants with two further types of prompts (specific and unspecific prompts) in a laboratory experiment. Finally, in Study 3, we varied the prompting instruction (detailed or general reflection instruction) in an online experiment.

## Study 1

Prilla et al. (2012, 2013a) have designed a web-based tool (the so-called “Talk Reflection App”) to support collaborative reflection in organizations and provide the staff with an instrument to share their work experiences. In a first use case the tool was designed to support medical staff of a hospital in reflecting on their conversations with patients’ relatives (Renner et al., 2014). This tool enables the externalization of one’s own experiences and allows for individual or collaborative reflection. The tool provides the opportunity for people to document experiences which they can reflect upon: others can add their own thoughts about the situations and specify the problem. Moreover, users can evaluate the outcomes and consider potential solutions.

As first experiences with the tool have shown, reflection on the experiences of others is not a trivial endeavor (Prilla, 2015). Response rates were low and people indicated that they sometimes were not sure how or what to reply to their colleagues. In order to foster participation and support individuals in collaborative reflection, the option for the implementation of prompts was integrated into the reflection tool (see Prilla and Herrmann, 2013). These prompts can be designed as questions that pop up while users read a problem description in the tool.

As outlined above, reflection includes the examination of a certain problem, but would ideally also result in conclusions that might provide suggestions for subsequent actions, that is, potential solutions to the problem. Therefore, we considered in the current study both the process of reflection about a given problem and the evaluation of the problem with regard to potential solutions. Accordingly, we implemented two types of prompts that were intended to specifically support these two different aspects. The objective of *problem prompts* was to foster the process of reflection by asking participants to analyze the given situation or to report similar experiences—without pointing to any potential future solutions. The objective of *solution prompts*, in contrast, was to lead to writing down possible advice in order to suggest how to handle the situation from that point on. So problem prompts related to the past and/or to the present situation, while solutions prompts referred to future activities. In line with the conceptual considerations presented above and with previous findings on the impact of prompting on reflection (e.g., Sobral, 2000; Van den Boom et al., 2004, 2007) we assumed that both types of prompts might have an effect on the quantity and quality (in terms of solution suggestions) of people’s contributions. Therefore, we stated the following hypotheses:

(H1.1) With prompts people will write more comprehensive comments than without prompts.
(H1.2) With prompts people will give advice that includes more suggestions for concrete solutions than without prompts.
(H1.3) With prompts people will give advice that includes more suggestions for general solutions than without prompts.

As an open research question we also considered potential differences between problem prompts and solution prompts with regard to the comprehensiveness of comments and the provision of advice that includes suggestions for concrete and general solutions.

### Material and Methods

#### Study Design

In this field study we applied a three-stage within-group design where we used the Talk Reflection App with participants from two organizations in the UK. We varied the prompts that participants received while they used the app: in half of the cases they did not receive any prompts while reading a problem description (control condition). In the other half of the cases they either received problem prompts (70% of the time) or solution prompts (30% of the time). (This unequal distribution of problem and solution prompts was due to the fact that the partners responsible for implementation were initially more interested in the analysis of problems than in solution suggestions. But this was not a problem for the study analysis since we did not analyze the absolute values but the number of words and suggestions per comment.)

As described above, problem prompts were given to encourage the process of reflection by inviting people to analyze the past and present of the problem described by their colleagues (why they arrived at this situation, what the current situation looked like). The following problem prompts were applied in this study: *“What could the author have done differently?,” “What are the reasons this has gone badly/well?,” “What changes would you suggest for similar situations?,” “Have you been in a similar situation? What did you do?,” “Do you want to know more about this situation?”* Solution prompts were given to lead the participants to write down suggestions for future activities (how to handle the situation from now on). The following solution prompts were applied: *“What can be learned from this situation?,” “Did you/your group come up with a solution of this/change for this?,” “Do you have an idea how to deal with this in the future?,” “What can be improved from this situation?,” “What has to be changed to improve similar situations?”* As the tool was used in actual practice during everyday work situations, prompts had to be short and easy to understand in order to stimulate answers without making it necessary to process too many resources. In addition to the original posts, the participants could also see other people’s replies.

#### Participants

Thirty employees of a public administration organization in the UK used the tool to reflect about work-related problems and questions. Additionally, a group of nine caregivers of a British care home for people suffering from dementia used the app to improve their skills in conversations with residents, relatives, and third parties. All participants used the app collaboratively, that is, the employees could share a problem with their colleagues who could then comment on it; those reflections were then in turn played back to the group.

#### Measures

As a basic indicator for people’s willingness to make contributions to the collaborative reflection process, we considered (1) the comprehensiveness of their comments in terms of the number of written words. In a second step we analyzed the content of the comments. For this purpose two raters examined how often the participants gave advice to their colleagues in terms of (2) concrete, situation-specific solution suggestions or (3) in terms of general solution suggestions.

Content was coded by two coders who initially discussed their appraisal of individual comments and came up with a joint decision. In the further course of the analysis, they examined the comments independently and we used the mean of the results of the two coders for analysis. Inter-rater agreement was acceptable for concrete solution suggestions with Krippendorff’s α = 0.73. For general solutions inter-rater agreement was not acceptable (Krippendorff’s α = 0.52; this problem remained even with another set of raters. This variable was therefore not considered for further analysis and Hypothesis 1.3 could not be tested in this study.

### Results

From the 139 comments extracted from the tool we had to exclude 21 from further consideration as they did not contain any topic-related content. While such content is to be expected and not unusual in a field setting, these aspects were not part of the focus of the present research. From the remaining 118 comments, 36 were written in the no prompt condition, 67 in the problem prompt condition, and 15 in the solution prompt condition.

Examples which illustrate concrete solution suggestions provided by employees of the public administration organization are presented in the two following statements: *“But from my experience of having to handle stressful calls, the key is to be calm – usually it’s the person on the other end getting angry, so just assert that you are not the reason for the problem, and if they’d like, you could perhaps transfer them to someone with more authority?” “I usually just tell my manager with an apology and reason and then ask if he wants to re-arrange it.”*

An analysis of variance (ANOVA) indicated differences in the comprehensiveness of comments among the three conditions, *F*(2,115) = 3.46, *p* = 0.035, $\eta_{p}^{2}$ = 0.06. Contrast analyses revealed that participants wrote longer comments in the solution prompt condition than in the problem prompt condition (*p* = 0.010) and, in line with Hypothesis 1.1, tended to write longer comments in the solution prompt condition than in the no prompt condition (*p* = 0.061).

Regarding concrete solution suggestions the ANOVA did not find significant differences among the three conditions, *F*(2,115) = 0.40, *p* = 0.674. Thus, Hypothesis 1.2 was not supported by the data. Means (*M*) and standard deviations (*SD*) of both dependent variables in the three conditions can be found in **Table 1**.

**Table 1:** Comprehensiveness of comments and concrete advice in the three conditions in Study 1.

	No prompt condition	Problem prompt condition	Solution prompt condition
	*M (SD)*	*M (SD)*	*M (SD)*
Number of words per comment	35.14 (26.62)	30.60 (23.01)	50.73 (40.57)
Concrete solution suggestions per comment	0.81 (0.72)	0.89 (0.83)	1.03 (1.09)

### Discussion

In this first study we found that when users were exposed to prompts that invited them to write down possible solutions, they wrote longer comments than when being confronted with prompts that asked them to analyze the problem or with no prompts at all. Regarding the content of the comments in the three conditions, we could not find any differences in the number of solution suggestions provided per comment. There may be several reasons for this unexpected finding. In such a field study there are many potential confounding variables and influences that cannot be entirely controlled.

For example, there were several different kinds of wording in the prompts and it is unclear what kind of reaction they elicited. It is also possible that the wording of the prompts was not sufficiently selective, that is, that problem prompts and solution prompts were not adequately targeted toward different aspects of the reflection process. In addition, the prompts sometimes were quite general in their wording. As a consequence, it is not clear to what extent the prompts provided by the tool fit the described experiences. While general wording of the prompts was necessary to increase the probability that prompts would fit a given situation, this might also be a reason for the lack of significant differences among the experimental conditions.

Another potential problem of this study was that participants belonged to two different groups, as they were recruited from two different organizations. In addition, these groups differed with respect to their size (*n* = 30 vs. *n* = 9). We cannot entirely rule out that group size or composition of the groups as well as other factors such as sex, age, or work experience had an impact on how they responded to the collaborative reflection scenario. Moreover, it is unclear whether the fact that participants could not only see the original post but also other people’s comments might have somehow diluted potential effects. In order to be able to eliminate a number of confounding variables, we proceeded in further studies with more controlled experimental settings. In particular, we made it a priority to accomplish a higher level of control for the impact of the particular wording of the prompts and for the background of the participants.

## Study 2

To research further the effects of prompts in an online reflection tool under more controlled conditions, we conducted a laboratory experiment with the Talk Reflection App. While controlling for external confounding variables, we still wanted to keep the setting as natural as possible. Therefore, we used the same tool, short prompts, and a realistic task for the participants. Again, we aimed to study whether prompts in general may increase people’s willingness to contribute more comprehensively to the collaborative reflection process. We also wanted to examine how to best support users in reflecting on problem descriptions and in providing (concrete as well as general) solutions to a given problem as described by another user.

From an applied perspective prompts would be considered to be particularly supportive if they are able to make participants reflect about the other users’ problems and specifically derive appropriate solution suggestions. In order to test whether such a *specific prompt*, asking for certain reflection elements, would be more effective than a more general, *unspecific prompt*, we set up pertinent experimental conditions. We stated the following hypotheses:

(H2.1) With prompts people will write more comprehensive comments than without prompts (re-examination of H1.1).
(H2.2) With specific prompts people will give advice that includes more suggestions for concrete solutions than with unspecific prompts or without prompts.
(H2.3) With specific prompts people will give advice that includes more suggestions for general solutions than with unspecific prompts or without prompts.

### Material and Methods

#### Study Design

Participants were invited to take part in an experiment about the discussion of problems at work or academic studies in online forums. They were asked to write down their own problematic situations in an initial step. Then, in the main part of the experiment, they were informed that other users had also already posted reports on similar problematic situations in the forum and that the participants should write comments regarding those posts. In this phase the study represented a three-stage between-group design with the between factor prompt (no prompt vs. specific prompt vs. unspecific prompt). Participants were randomly assigned to one of the three conditions.

In the no prompt condition participant worked in a forum with five existing problem descriptions each with a standard comment field at the bottom. In contrast to the previous study, there was only one particular prompt in each of the two prompt conditions. In the unspecific prompt conditions participants worked in the same forum with the same five problem descriptions, but when they clicked on a description to read it, a prompt popped up that provided the following text: *“Is something coming to your mind regarding this situation?”* In the specific prompt condition the prompt asked the participants explicitly to specify the problem and to provide possible ways to react to that problem: *“Which problem pattern is reflected here, which way to react comes to your mind?”*

Participants were assembled in groups of up to six persons who worked parallel on individual notebooks in one room. These participants did not work together, however; they were only participating at the same time in the same experiment, but did so independently. After the introduction participants worked in the forum for half an hour. Following the work in the forum they answered a questionnaire on the computer in order to evaluate the prompts. Finally, participants were paid eight Euros for participation, debriefed, and dismissed.

#### Participants

Seventy-eight university students participated in this experiment. Ten participants in the two prompt conditions indicated afterward that they had not noticed any prompts (treatment check). These participants were removed from the data before analysis. This procedure resulted in 26 participants in the control condition without prompts, 23 in the unspecific prompt condition, and 19 in the specific prompt condition. 47 of them were women and 21 were men. The participants’ mean age was *M* = 24.65 years (*SD* = 5.78).

#### Measures

As in Study 1 we counted the number of words per comment. We also coded the concrete and general solution suggestions in the comments. The inter-rater agreement was good for concrete solutions (Krippendorff’s α = 0.87) and acceptable for general solutions (Krippendorff’s α = 0.67).

### Results

The following statements are examples of *concrete* solution suggestions provided by participants (translated by the authors): *“For me, in such a situation it is helpful to prepare myself thoroughly and to acquire information about the institution of the internship.” “I would recommend going to the post-exam review.”*

Examples of *general* solution suggestions are presented in the following statements: *“I think you should behave more self-confidently.” “I suppose you have to work in a very structured way in order to manage that somehow.” “Don’t cross your bridges before you come to them.”*

When comparing the mean number of words per comment we found a significant effect of prompts, *F*(2,65) = 3.81, *p* = 0.027, $\eta_{p}^{2}$ = 0.11. In contrast to the assumptions stated in Hypothesis 2.1, we found that participants wrote significantly less in the unspecific prompt condition than in the other two conditions (quadratic contrast: *p* = 0.010).

Regarding the concrete solution suggestions, we found that participants in the no prompt condition provided advice that included significantly more suggestions for concrete solutions per comment than participants in the other two conditions, *F*(2,65) = 2.77, *p* = 0.070; quadratic contrast: *p* = 0.022, $\eta_{p}^{2}$ = 0.08. Thus, Hypothesis 2.2 was not supported by the data. For general solution suggestions we found no significant differences among the three groups, *F*(2,65) = 0.28, *p* = 0.754. Accordingly, Hypothesis 2.3 was not supported by the data either. Means (*M*) and standard deviations (*SD*) of the three dependent variables in the three conditions are shown in **Table 2**.

**Table 2:** Comprehensiveness of comments and concrete and general advice in the three conditions in Study 2.

	No prompt condition	Specific prompt condition	Unspecific prompt condition
	*M (SD)*	*M (SD)*	*M (SD)*
Number of words per comment	94.81 (39.44)	88.47 (37.97)	66.13 (34.80)
Concrete solution suggestions per comment	1.73 (0.89)	1.26 (0.57)	1.31 (0.74)
General solution suggestions per comment	1.30 (0.53)	1.26 (0.60)	1.16 (0.78)

Since the results did not quite reveal the pattern we had expected, we conducted further analyses to shed some light on our findings. In the questionnaire people answered after they had worked in the forum, participants of the two prompting conditions were asked to evaluate the prompts, for example, whether they liked them, if they perceived them as disturbing etc. We analyzed these comments and found that participants in the specific prompt condition indicated to have been disturbed or confused by the prompt significantly more often than participants in the unspecific prompt condition (seven of 19 comments vs. zero of 18 comments, Fisher Exact Probability Test: *p* = 0.005, Inter-rater agreement: Krippendorff’s α = 0.75).

### Discussion

In this lab study we found not only an effect of prompts on the length of comments but also on the content. However, our hypotheses were not supported by the data. Comments in the unspecific prompt condition were even shorter than in the no prompt condition. An explanation for the lack of suitability of the specific prompt could be that this prompt, although quite short, was not ideal in terms of comprehensibility. As some participants mentioned in the open answers, they tended to be disturbed by the specific prompt or to find it artificial as the term “problem pattern” was not very helpful for them. So we conducted an additional experiment where we paid close attention to finding wording for the prompts that spelled out what we meant by “problem pattern” and that we thus considered to be more beneficial and acceptable to the users.

## Study 3

As the second study had indicated that users were dissatisfied with short, abstract prompts we conducted a third study using *detailed instructions* in order to help participants reflect on problem descriptions and write helpful comments. In this study we focused on the reflection instructions provided by a prompt and did not use the tool that we had employed in the previous studies. In doing so we aimed to avoid participants being irritated by not knowing how to use the tool or becoming distracted by the pop-up windows. In line with these considerations and our previous reasoning we expected the detailed reflection instructions would be beneficial to the quantity and quality of people’s contributions. Accordingly, we stated the following hypotheses:

(H3.1) With detailed reflection instructions people will write more comprehensive comments than with global reflection instructions.
(H3.2) With detailed reflection instructions people will give advice that includes more suggestions for concrete solutions than with global reflection instructions.
(H3.3) With detailed reflection instructions people will give advice that includes more suggestions for general solutions than with global reflection instructions.

### Material and Methods

#### Study Design

Participants were invited to answer an online questionnaire in which they found four problems of fictitious fellow students. Participants were asked to help those fellow students by writing comments on their problems that were described in brief texts of 52–73 words. An example illustrating such a problem is the following statement (translated by the authors):

“I am completely stressed out. Next week I will have to give two seminar presentations and submit a report. Actually I wanted to start on it last week, but then I had to help my parents with their restaurant, because my father has broken his leg. Unfortunately, I cannot postpone anything, because the dates have already been fixed for a long time. Somehow I have to handle it all.”

The experiment was a between-group design with the two-stage factor *instructions*. One half of the participants served as a control group with general reflection instructions. They were merely asked to help the fictitious other students with their problems by reflecting on them and writing an answer. The other half of the group received detailed instructions about how to reflect on the problems. These detailed reflection instructions followed previous research on interpersonal knowledge transfer with patterns, that is, pre-structured templates that address both problems and solutions (e.g., Bokhorst et al., 2014; Moskaliuk et al., 2016):

“Please help the person by reflecting on her problem and giving her an answer in the open text field. Please consider in your answer the following aspects:

1. Have you been in a similar situation and what were your experiences with it? (thoughts, feelings, behavior)
2. Why did you act like you did and would you act differently now in hindsight?
3. What helped you in your situation and what problems did you have?
4. Can you imagine how to transfer your solution to other situations? If so, which situations?”

The study was conducted as an online study and participants were distributed randomly to the two conditions. Participation was on a voluntary basis and not paid. Participants first reflected on the four different hypothetical problems of other students and then answered a questionnaire with demographics and a treatment check.

#### Participants

Sixty university students participated in this study, 32 students were assigned to the control condition, 28 to the detailed reflection instructions condition. Eight participants had to be removed from the detailed reflection condition after failing the treatment check. Of the remaining participants 31 were women, 18 were men (and three persons did not indicate their sex and age). Participants’ mean age was *M* = 24.53 years (*SD* = 5.82).

#### Measures

Once more, we captured the comprehensiveness of comments in number of written words and coded the content of the replies regarding concrete and general solution suggestions. Inter-rater agreement was acceptable for both concrete (Krippendorff’s α = 0.78) and general solution suggestions (Krippendorff’s α = 0.70).

### Results

Again, we first looked at the length of comments in terms of words per comments. In line with Hypothesis 3.1 we found significantly longer comments in the detailed instructions condition than in the control condition, *t*(50) = 2.77, *p* = 0.008, *d* = 0.79.

With regard to content, there were no differences in concrete solution suggestions between the two groups, *t*(50) = 1.08, *p* = 0.287. But we found that participants in the detailed reflection instructions condition provided significantly more general solution suggestions per comment than the participants in the control condition as assumed in Hypothesis 3.3, *t*(25.97) = 3.00, *p* = 0.006, *d* = 0.86. Means (M) and standard deviations (SD) of the three dependent variables in the detailed and the global reflection condition are shown in **Table 3**.

**Table 3:** Comprehensiveness of comments and concrete and general advice in both conditions in Study 3.

	Detailed reflection instructions	Global reflection instructions
	*M (SD)*	*M (SD)*
Number of words per comment	110.78 (50.29)	70.22 (52.05)
Concrete solution suggestions per comment	1.57 (0.89)	1.29 (0.93)
General solution suggestions per comment	2.10 (1.10)	1.30 (0.59)

### Discussion

In this study we found that detailed reflection prompts helped participants to reflect on other people’s problems: they wrote longer comments overall and generated more general solution suggestions. We did not find any differences in the number of concrete solution suggestions, however. It seems that giving concrete advice to someone with a particular problem is quite common and happens naturally independently of any particular instructions. To reflect about the underlying problem, however, and to elaborate more deeply upon more general solution suggestions, which might be particularly helpful for other situations as well, apparently needs further support. In this third study we could show that it is possible to increase the provision of general solution suggestions with detailed reflection instructions.

## General Discussion

Taken together, our insights from three experiments on fostering collaborative reflection with different types of prompts indicate that one can affect user behavior in an online reflection tool with the help of particular instructions. However, one important aspect is that the wording of such a prompt has to be well chosen. It needs to be adjusted to the target group of the tool. We found, for example, that participants in the specific prompt condition in Study 2 indicated that they had been disturbed or confused by this prompt that asked them to reflect on a “problem pattern” without sufficiently specifying what exactly that was supposed to be. It is possible, though not verifiable from the data, that such confusion might have also been a problem in the prompt conditions in Study 1.

Additionally, results of our third study indicate that detailed, easily understandable instructions may help people to write reflective comments on problematic experiences of others by introducing more general solution suggestions. Providing general advice can be particularly valuable since solutions that are not only applicable to a concrete, narrowly defined situation but also relevant to a broader range of situations might be helpful for more people (Bokhorst et al., 2014; Moskaliuk et al., 2016). When evaluating the suitability of prompts for supporting collaborative reflection we should also keep in mind that the effect sizes that we have reported were quite low in all three studies. In addition, the high standard deviations for all of the dependent variables indicated that there seemed to be a large inter-individual variance in people’s willingness to contribute to collaborative reflection. Moreover, it is quite likely that the particular type and purpose of a tool or platform have a strong impact on people’s willingness to make contributions.

For research into and practice of computer-supported collaborative reflection, our results could mean that developers should not only aim at integrating scaffolding tools such as prompts but should also make sure that users get comprehensive instructions on how to reflect when they start using such a tool. Another idea could be to integrate instructions into the tool itself and provide an area in the tool where users may get some advice if necessary. Prompts could, for example, pop up only after a certain latency time in which a user has not yet started to write a comment and offer some advice at that time. Users could click on a link in the prompt to see some detailed information with several different aspects that could be considered when reflecting on a problematic situation. In this way, disturbing or annoying users with prompts could be avoided and instead just-in-time advice could be provided at the moment when they really need it.

The relatively heterogeneous pattern of results across our three studies indicates that it is important to get an overall picture by examining the phenomenon in different settings, both in the lab and in the field. In the studies presented here, for practical reasons, we had to use different types of participant samples. Future studies should aim to examine various prompts with various wording with the same type of sample in order to exclude alternative explanations for their findings that could be attributed to the sample rather than the experimental treatment. Future studies could also include further dependent variables, such as measures for deep levels of reflection or indices for other aspects of the reflection process. In any case, further research should keep an eye out for the possibility of finding a balance between a high level of experimental control over potentially influencing factors and confounding variables on the one hand, and the need for a high level of ecological validity on the other hand. Both are required for the examination of such application oriented research questions in organizational psychology.

## Author Contributions

BR, MP, UC, and JK made substantial contributions to the conception and design of the work; BR and JK were involved in the analysis and interpretation of data for the work. BR and JK drafted the work; MP and UC revised it critically for important intellectual content. BR, MP, UC, and JK approved the final version to be published. BR, MP, UC, and JK agree to be accountable for all aspects of the work in ensuring that questions related to the accuracy or integrity of any part of the work are appropriately investigated and resolved.

## Conflict of Interest Statement

The authors declare that the research was conducted in the absence of any commercial or financial relationships that could be construed as a potential conflict of interest. The research reported here was funded by a grant of the European Commission. This sponsor did not exert influence on the content of this research in any way.
